# Editorial: Metabolic pathways in early embryogenesis: mechanisms and implications

**DOI:** 10.3389/fcell.2026.1924590

**Published:** 2026-07-24

**Authors:** Marcella Pecora Milazzotto, Marcos Roberto Chiaratti, Jo Leroy, Camila Bruna de Lima

**Affiliations:** 1 Center of Natural and Human Science, Federal University of ABC, Santo André, Brazil; 2 Departamento de Genética e Evolução, Centro de Ciências Biológicas e da Saúde, Universidade Federal de São Carlos, São Carlos, Brazil; 3 Department of Veterinary Sciences, Gamete Research Center, Veterinary Physiology and Biochemistry, Universiteit Antwerpen, Antwerp, Belgium; 4 Département des Sciences Animales, Centre de Recherche en Reproduction, Développement et Santé Intergénérationnelle (CRDSI), Université Laval, Québec City, QC, Canada

**Keywords:** embryo, gamete, metabolism, molecular dynamic, reproduction

From the moment of fertilization, the mammalian embryo begins a developmental trajectory that is metabolically demanding and molecularly complex. The transition from a fertilized oocyte to a competent blastocyst requires tight coordination of nutrient sensing, energy production, redox balance, and biosynthetic fluxes with the molecular programs that govern genome activation, lineage specification, and implantation. The contributions assembled in this Research Topic, “*Metabolic Pathways in Early Embryogenesis: Mechanisms and Implications,*” collectively underscore that metabolism is not a passive background process but a primary driver and regulator of early development, with implications for implantation, fetal health, and assisted reproduction.

A central theme emerging from this Research Topic is that metabolic pathways are linked to the formation and functional specialization of the earliest embryonic lineages. Khan et al. identify carbohydrate-responsive element–binding protein (ChREBPα) as a key metabolic sensor coordinating lipid droplet (LD) biogenesis during mouse preimplantation development. Mechanical delipidation of zygotes followed by culture under fatty-acid–free conditions reveal a striking capacity for rapid LD regeneration, driven by upregulation and nuclear translocation of ChREBPα and *de novo* lipogenesis. Pharmacological inhibition of ChREBP compromises LD renewal and results in developmental arrest at the morula–blastocyst transition, pointing to this stage as a critical metabolic bottleneck in which trophectoderm (TE) formation is particularly vulnerable. These findings position ChREBPα at the center of lipid storage, and lineage-specific energy allocation, and reinforce the concept that LDs are not inert fat deposits but dynamic organelles essential for blastocoel formation and TE differentiation.

The notion that metabolic pathways drive the progression to blastocyst is further strengthened by Berling et al., who focus on mechanistic target of rapamycin (mTOR) signaling as a master integrator of nutrient availability and growth in bovine embryos. Their work demonstrated that mTOR signaling exhibits a distinct spatial and temporal activation pattern during early bovine embryo development, closely associated with trophectoderm (TE) differentiation. While mTOR protein was detected in both the cytoplasm and nucleus of morulae, its localization became predominantly nuclear in blastocysts. Pathway activity, assessed by phosphorylated S6 (pS6), was primarily detected in the outer cells of morulae and subsequently in the TE of early blastocysts, declining progressively as embryos developed into expanded blastocysts. More advanced blastocysts exhibited lower pS6 levels, suggesting a reduction in mTOR activity during later stages of preimplantation development. These findings indicate that mTOR signaling is preferentially activated in cells committed to the TE lineage, supporting the metabolic and translational programs required for the first cell differentiation event in bovine embryos.

Metabolism also emerges as a critical determinant of growth factor signaling and morphogenesis. Park et al. demonstrated that insulin-like growth factor 1 receptor (IGF1R) signaling is essential for regulating blastocyst developmental kinetics, expansion, and survival in porcine embryos, although it is dispensable for early cleavage divisions and cell lineage specification. Genetic ablation of IGF1R delayed the morula-to-blastocyst transition, reduced total cell number, and increased apoptotic susceptibility without disrupting trophectoderm, epiblast, or primitive endoderm lineage segregation. Pharmacological inhibition of IGF1R/INSR recapitulated this phenotype, indicating that insulin receptor signaling does not compensate for the loss of IGF1R during preimplantation development. Furthermore, IGF-1 supplementation failed to rescue the developmental defects caused by receptor inhibition or deletion and instead exacerbated apoptotic responses when receptor signaling was compromised. Collectively, these findings establish IGF1R signaling as a key regulator of blastocyst growth, proliferation, and embryonic viability, acting primarily by controlling developmental progression rather than cell fate specification.

The work by Dilixiati et al. employed an integrated multi-omics approach to uncover the molecular adaptations of sheep oocytes to vitrification, identifying proline as a key protective metabolite against cryoinjury. The authors showed that vitrification induces extensive metabolic and transcriptomic reprogramming, with increased intracellular proline levels driven by the upregulation of PYCR3 and downregulation of P4HA1, highlighting arginine and proline metabolism as one of the most significantly enriched pathways. Guided by these findings, supplementation of the vitrification medium with 0.5 M L-proline significantly improved post-thaw oocyte survival and developmental competence by reducing oxidative stress, preserving spindle integrity, mitochondrial and endoplasmic reticulum organization, maintaining mitochondrial membrane potential, ATP production, and calcium homeostasis. Collectively, this study identifies proline as both an endogenous adaptive metabolite and a promising therapeutic additive for improving oocyte cryopreservation outcomes.

A second major theme is the extension of metabolic regulation beyond the embryo proper to encompass endometrial receptivity and the peri-implantation interface. The work by Zhang et al. provides a comprehensive review highlighting the emerging parallels between the Warburg effect and endometrial receptivity, proposing that embryo implantation relies on metabolic adaptations similar to those observed in rapidly proliferating cancer cells. The authors discuss evidence that blastocysts and trophoblasts establish a lactate-rich, acidic microenvironment through aerobic glycolysis, which promotes endometrial receptivity by modulating immune tolerance, cytokine signaling, epigenetic regulation, and trophoblast invasion. The review further integrates current knowledge on the roles of glycolytic metabolism, mitochondrial dynamics, histone lactylation, and hormone-dependent regulation in coordinating embryo–endometrium communication. Although direct experimental evidence remains limited, this work presents a compelling conceptual framework linking metabolic reprogramming to implantation success and identifies the Warburg-like metabolic state as a promising target for improving endometrial receptivity and reproductive outcomes.

If metabolism shapes not only the embryo and uterus but also our capacity to assess and select viable embryos, then there is a pressing need for robust, non-invasive metabolic analysis. This need is addressed by Cao et al., who present an original study combining Raman spectroscopy of day-3 spent culture medium with machine learning to predict the capacity of cleavage-stage human embryos to form good-quality blastocysts. Using Raman metabolic fingerprints from Day 3 culture media samples, the authors trained and compared twelve machine learning models to classify embryos according to their extended culture outcomes: morphologically good blastocysts, morphologically non-good blastocysts, or clinically non-useful embryos. A multilayer perceptron (MLP) achieved the best individual performance, while an ensemble stacking strategy further improved prediction, reaching an overall accuracy of 94%, with 93% sensitivity and 97% specificity. By integrating complex metabolic signatures rather than relying solely on embryo morphology, this approach provides an objective assessment of embryo quality and may allow reliable selection of high-potential embryos for transfer at the cleavage stage, potentially reducing the need for extended blastocyst culture and its associated risks. Although promising, the authors emphasize that larger, multicenter validation studies and randomized clinical trials are required before routine clinical implementation.

This Research Topic illustrates how diverse approaches can be brought together to point to metabolism as a common principle in early embryogenesis. These articles collectively pave the way toward more physiologically informed embryo culture systems, better predictors of developmental competency, and improved reproductive outcomes.

We hope that this Research Topic will stimulate further interdisciplinary research at the interface of developmental biology, metabolism, reproductive medicine, and systems biology, and that it will help to shift the field from viewing metabolism as a background “support service” to recognizing it as a central actor of early life.

